# Improve the Deep Learning Models in Forestry Based on Explanations and Expertise

**DOI:** 10.3389/fpls.2022.902105

**Published:** 2022-05-19

**Authors:** Ximeng Cheng, Ali Doosthosseini, Julian Kunkel

**Affiliations:** ^1^Gesellschaft für wissenschaftliche Datenverarbeitung mbH Göttingen, Göttingen, Germany; ^2^Department of Artificial Intelligence, Fraunhofer Heinrich Hertz Institute, Berlin, Germany

**Keywords:** explainable artificial intelligence, forest care, deep neural networks, feature unlearning, classification

## Abstract

In forestry studies, deep learning models have achieved excellent performance in many application scenarios (e.g., detecting forest damage). However, the unclear model decisions (i.e., black-box) undermine the credibility of the results and hinder their practicality. This study intends to obtain explanations of such models through the use of explainable artificial intelligence methods, and then use feature unlearning methods to improve their performance, which is the first such attempt in the field of forestry. Results of three experiments show that the model training can be guided by expertise to gain specific knowledge, which is reflected by explanations. For all three experiments based on synthetic and real leaf images, the improvement of models is quantified in the classification accuracy (up to 4.6%) and three indicators of explanation assessment (i.e., root-mean-square error, cosine similarity, and the proportion of important pixels). Besides, the introduced expertise in annotation matrix form was automatically created in all experiments. This study emphasizes that studies of deep learning in forestry should not only pursue model performance (e.g., higher classification accuracy) but also focus on the explanations and try to improve models according to the expertise.

## 1. Introduction

Due to climate change, environmental damage, and other related factors, extreme weather events (e.g., wildfires, heat waves, and floods) are occurring more frequently all over the world in recent years (Stott, 2016). As essential cogs in the global ecosystem, forests have many ecological functions including conserving water, protecting biodiversity, and regulating climate (Führer, 2000; Zhang et al., 2010). Therefore, forest care is vital for our future. Fortunately, the United Nations has proposed 17 *Sustainable Development Goals*, where the 13th goal *climate action*, and 15th goal *life on land* pertain to forest care^1^. This has promoted studies in forestry.

*Remote sensing* technology has provided data with high spatio-temporal resolution and many spectral bands for forestry research, which allows researchers to use more information to build a model than traditional ways of collecting data in the wild. Due to the ability to gain knowledge from large amounts of train data, artificial intelligence technology represented by *deep learning* models has also been applied in forestry to accomplish diverse tasks (Wang et al., 2021) including tree species classification (Wagner et al., 2019) and damage assessment (Hamdi et al., 2019; Tao et al., 2020). In terms of the data types, most studies in forestry have used deep learning models to analyze remote sensing data (Zhu et al., 2017; Diez et al., 2021), such as unmanned aerial vehicle (UAV) data (Diez et al., 2021; Onishi and Ise, 2021), high-resolution satellite images (Li et al., 2017), and 3-D point cloud data (Zou et al., 2017). There are also some studies based on other data types including the images of digital cameras (Liu et al., 2019) and the characteristics of individual trees (Ercanlı, 2020). Deep learning models are regarded as black boxes due to their complicated network structures and a large number of parameters (Castelvecchi, 2016). Although trained models can achieve excellent performance, it is difficult for researchers and users to understand how they make decisions. This indicates that the model may not have gained the correct knowledge (e.g., Clever Hans^2^), and also undermines the users' confidence in the deep learning models.

To interpret the black-box models, researchers focus on the studies of *explainable artificial intelligence* (XAI) methods (Samek et al., 2019). Many XAI methods with different principles have been proposed and can be divided into three categories: *visualization methods, model-agnostic methods*, and *deep-learning-specific methods*. The first category consists of new visualization methods to display the parameters of complex models (e.g., random forests and neural networks) (Zeiler and Fergus, 2014; Zhao et al., 2018), such as clustering the original model parameters or displaying feature maps of part layers. *Model-agnostic methods* can be used to interpret any model because these methods only consider the variation of model outputs following perturbing inputs (Ribeiro et al., 2016b; Molnar, 2020). Common model-agnostic approaches include individual conditional expectation (ICE) (Goldstein et al., 2015), local interpretable model-agnostic explanations (LIME) (Ribeiro et al., 2016a), and Shapley additive explanations (SHAP) (Lundberg and Lee, 2017). Besides, some studies have proposed advanced model-agnostic approaches to combine the local explanations (i.e., sample-based) and global explanations (i.e., feature/variable-based) (Giudici and Raffinetti, 2021). The *deep-learning-specific methods* such as layer-wise relevance propagation (LRP) (Bach et al., 2015) and gradient-weighted class activation mapping (Grad-CAM) (Selvaraju et al., 2017) are designed to interpret trained deep learning models based on detailed network information (e.g., gradients). These methods are typically used to get sample-based explanations in image classification tasks. Several studies use multiple XAI methods to interpret trained models, such as using Grad-CAM to obtain the contributions of input pixels as well as visualizing the feature maps of part layers (Xing et al., 2020). In addition to computer science, XAI methods have been applied in various fields including medicine (Tjoa and Guan, 2020), geography (Cheng et al., 2021), and disaster assessment (Matin and Pradhan, 2021). However, few studies have attempted to interpret models in the field of forestry (Onishi and Ise, 2021), even though deep learning methods have been widely applied in this field.

XAI methods provide explanations of deep learning models, but this is not sufficient for practical purposes. For specific tasks, researchers wish to guide the training based on expertise in a way that the models gain the correct knowledge (i.e., what we believe the model should learn) and avoid the Clever Hans effect (Lapuschkin et al., 2019; Anders et al., 2022). The approaches used to guide the training of deep learning models are known as *feature unlearning* (FUL) methods, and these methods utilize one of two main ideas: The first idea is perhaps the most direct, in which models are retrained with reformed train data (e.g., explanatory interactive learning (XIL); Teso and Kersting, 2019; Schramowski et al., 2020). For instance, if some error-prone samples are affecting the model's performance, it can be improved by simply removing these samples from the train data and then retraining the model. The second idea is to design a new loss function to highlight the weight of important features according to expert knowledge, such as adding a mask to mark useless pixel areas in image classification tasks. The common methods with this idea include right for the right reasons (RRR) (Ross et al., 2017) and contextual decomposition explanation penalization (CDEP) (Rieger et al., 2020). Several more complicated methods exist such as learning not to learn (LNTL) (Kim et al., 2019). In addition to using new loss functions and retraining models based on new train data, LNTL also alters the network structure. Many FUL methods have been proposed, but most are not commonly used in practice. In this study, we will apply FUL methods in the field of forestry.

This study aims to improve the deep learning models in forestry based on the obtained model explanations and specialized expertise. Deep learning models can mine massive amounts of original data. XAI methods can shed light on the black boxes and provide explanations. If the explanations are not as expected, FUL methods can be used to guide the training and improve the credibility and performance of deep learning models. The main contributions of this paper can be summarized as: (1) using explanations and expertise to improve deep learning models, which is the first such attempt in the field of forestry; (2) emphasizing that explanations reflect how the model make decisions, which is vital for black-box models; (3) a new research framework is proposed and serves as a reference for deep learning studies in forestry.

The paper is organized as follows: Section 2 describes the proposed research framework and the basic principles of applied Grad-CAM and RRR methods. We also introduced three indexes to assess the model explanations. To verify this study, three experiments based on simulated data and real data were carried out in Section 3. The results show that the model accuracy can be improved and the explanations can be altered as expected. Section 4 discusses the impact of outlier data and sampling variability on model performance. We summarized this research and provided future directions in Section 5.

## 2. Methods

### 2.1. Research Frameworks of Deep Learning Studies

**Figure 1:**
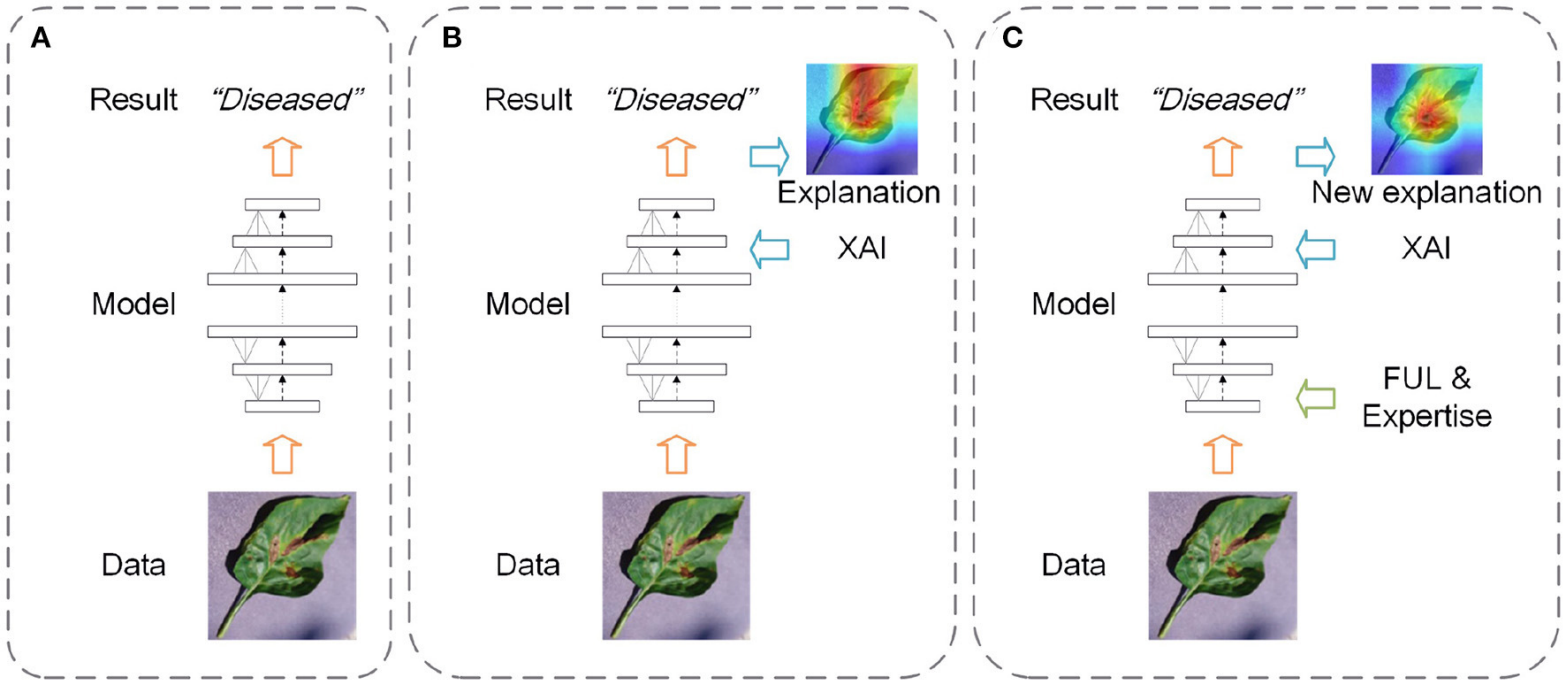
Three frameworks of deep learning studies (**A**: get the results only; **B**: get the results and explanations; **C**: improve the model based on explanations and expertise).

In common studies that utilize deep learning models to accomplish tasks, the focus is mostly on achieving higher performance rather than making sure that the trained models make decisions properly (i.e., black-box models) (as displayed in Figure 1A). In further studies, XAI methods have been applied to explain the trained models and obtain the explanations corresponding to the results (as displayed in Figure 1B). Based on the explanations and expertise, researchers can judge whether the trained models have gained the correct knowledge from the data. In this article, we propose a new research framework (as displayed in Figure 1C). It has four steps including training an original model, getting the model explanations, introducing the expertise based on the current explanations, and retraining the model with the introduced expertise. The FUL methods are used to guide the training when the original model explanations are inconsistent with expertise. Compared to the other two frameworks, the framework of our research is not only pursuing the model performance but also using explanations and expertise to interpret and improve the deep learning models. In this study, we select the image classification tasks in forestry as the specific application of the proposed research framework.

### 2.2. The Applied XAI Method: Gradient-Weighted Class Activation Mapping

This research uses the Grad-CAM method (Selvaraju et al., 2017) to obtain the corresponding explanations of each input (i.e., intuitive visualization of pixel importance) from the trained deep learning models. Grad-CAM is a prominent XAI method that has been applied extensively in computer vision tasks. Considering that all three experiments in this research are based on image data, we take the reliable Grad-CAM method to represent XAI methods and do not discuss others nor their differences in resulting explanations. Grad-CAM is based on the class activation map (CAM) methods (Zhou et al., 2016). It uses the gradient information in the training process to determine the neurons' importance in the model's decision, i.e., the neurons with larger absolute values of gradients are more important.

**Figure 2:**
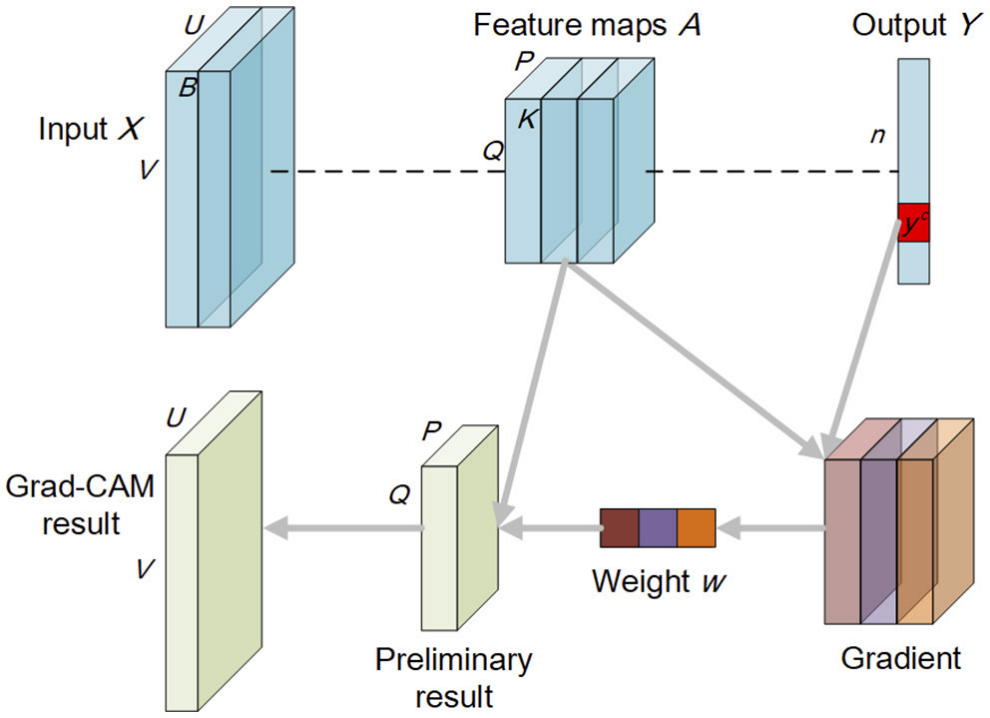
Schematic diagram of the Grad-CAM method.

Given *M* as the trained neural network, *X* ∈ ℝ^*U*×*V*×*B*^ as the input image with width *U*, height *V*, and *B* bands, *A* as the feature maps with width *P*, height *Q*, and *K* bands (i.e., *A*^1^, *A*^2^, …, *A*^*k*^) in the last convolutional layer, *Y* = [*y*^1^, *y*^2^, …, *y*^*n*^] as the output variable before the softmax in a n-classification task, $\frac{\partial y^{c}}{\partial A}$ denotes the gradient corresponding to class *c*, Equations (1) and (2) represent the formula for the Grad-CAM explanations [i.e., *G*(*M, X, c*)]. Figure 2 also illustrates the Grad-CAM method.


(1) $$\begin{array}{l} w_{k}^{c}=\frac{1}{P\times Q}\overset{P}{\underset{i}{\sum }}\overset{Q}{\underset{j}{\sum }}\frac{\partial y^{c}}{\partial A_{ij}^{k}} \end{array}$$

(2) $$\begin{array}{l} G\left(M,X,c\right)=Trans\left(ReLU\left(\overset{K}{\underset{k}{\sum }}w_{k}^{c}A^{k}\right)\right), \end{array}$$

where $\frac{1}{P\times Q}\overset{P}{\underset{i}{\sum }}\overset{Q}{\underset{j}{\sum }}$ denotes the global average pooling process, $w_{k}^{c}$ denotes the weight of feature map *k* corresponding to class *c* in the linear combination. *ReLU*(·) is placed to only consider features that have a positive impact on classification. It is noted that the preliminary Grad-CAM explanations are of the same size as the feature maps *A* (i.e., *P* × *Q*). Thus, need to use the *Trans*(·) function to transform them into the size of inputs (i.e., *U* × *V*).

### 2.3. The Applied FUL Method: Right for the Right Reasons

This research selects the RRR method (Ross et al., 2017) as an example of many FUL methods to improve deep learning models based on expertise. The basic idea of RRR is to add another *right reason loss* (RRR loss) into the common loss function (e.g., cross-entropy) and guide the model training. As mentioned in the Grad-CAM method introduction, the gradient information of variables reflects their influences on the deep learning model. The new loss aims to reduce the input gradient of useless pixels identified by the annotation matrix of each sample and drive the model to focus on the important features according to expertise. The annotation matrix can be viewed as a binary mask that splits pixels into two parts for the specific task. Zero-element and one-element label the useful pixels and useless pixels, respectively.

According to the experimental results, the original RRR formula (Ross et al., 2017) has been altered in this research. Given θ as the model parameters, *X*_*i*_ as an input image, *Y*_*i*_ as the model output of *X*_*i*_, *A*_*i*_ as the corresponding annotation matrix with the same size as inputs, the new loss function using the RRR method (i.e., *NLoss*) can be represented by Equations (3)–(6).


(3) $$\begin{array}{l} Grad_{X_{i}}=\frac{\partial \log_{\text{e}}\left(Y_{i}+1\right)}{\partial X_{i}} \end{array}$$

(4) $$\begin{array}{l} RLoss\left(X_{i},Y_{i},\theta ,A_{i}\right)=Sum\left(A_{i}\cdot Grad_{X_{i}}\right) \end{array}$$

(5) $$\begin{array}{l} NLoss=CLoss+\lambda \cdot Balance\left(RLoss,CLoss\right) \end{array}$$

(6) $$\begin{array}{l} Balance\left(l_{1},l_{2}\right)=10^{\left\lceil \log_{10}\left(\frac{l_{2}}{l_{1}}\right)\right\rceil }\cdot l_{1}, \end{array}$$

where *Grad*_*X*_*i*__ denotes the gradient of input *X*_*i*_ in the training process. *RLoss* is the added loss. *Sum*(·) is the function to sum all the elements of the controlled gradient *A*_*i*_ · *Grad*_*X*_*i*__. *CLoss* is the common loss such as cross-entropy. *Balance*(·) is the function to control the values of two losses in an order of magnitude. λ is the weight of the RRR loss in model training.

**Figure 3:**
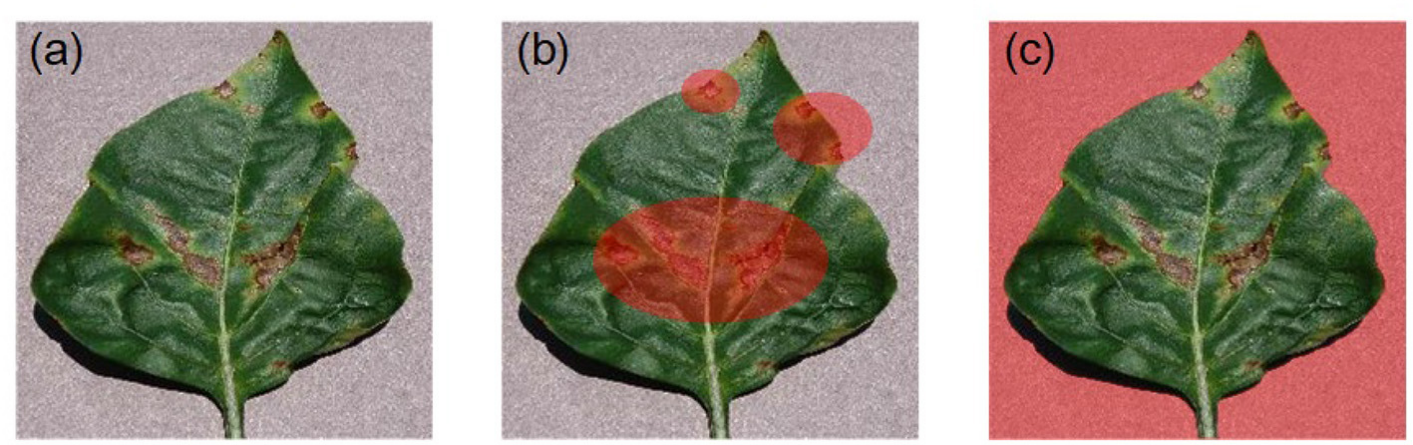
An example of diseased leaves and the corresponding masks (i.e., annotation matrix) (**a**: original image; **b**: labeling the important bacterial spots; **c**: labeling the useless background). The labeled pixels are red.

The annotation matrix is critical for guiding model training. In practical applications of forestry, it is difficult to set the annotation matrix of each sample due to the huge amounts of train data and the required expertise. For the RRR method, the annotation matrix of some samples can be set as a zero matrix. In this case, the loss function for model training will essentially reduce to the common one. Besides, the annotation matrix is used to label the useless area, which is easier than labeling the important features and increases the robustness of mask setting (e.g., just label unquestionably useless pixels such as the background). Take the task of identifying diseased leaves as an example (displayed in Figure 3). The bacterial spots in a leaf are labeled in Figure 3b depending on expertise. But the labeling is difficult to accomplish automatically and avoid omissions. In comparison, the useless background pixels for this task are labeled in Figure 3c by simple image processing (e.g., background extraction).

### 2.4. Explanation Assessment

This research aims to guide the training of deep learning models based on expertise. It manifests as better model performance and closer explanations to the predetermined *real masks* (i.e., annotation matrix). We use three indicators, root-mean-square error (RMSE)^3^, cosine similarity (CosineS)^4^, and the proportion of important pixels (PIP) labeled in real masks, to assess the obtained explanations from three aspects including absolute difference, relative difference, and differences in key features.

Given *A* = [*a*_1_, *a*_2_, …, *a*_*N*_] as an obtained explanation, *B* = [*b*_1_, *b*_2_, …, *b*_*N*_] as the real mask with the same size of *A*, Equations (7) to (9) represent three indicators of explanation assessment.


(7) $$\begin{array}{c} \text{RMSE}=\sqrt{\frac{\sum_{i=1}^{N}{\left(a_{i}-b_{i}\right)}^{2}}{N}} \end{array}$$

(8) $$\begin{array}{c} \text{CosineS}=\frac{\sum_{i=1}^{N}a_{i}b_{i}}{\sqrt{\sum_{i=1}^{N}a_{i}^{2}}\sqrt{\sum_{i=1}^{N}b_{i}^{2}}} \end{array}$$

(9) $$\begin{array}{l} \text{PIP}=\frac{Num\left(\text{IP}\cap \text{RM}\right)}{Num\left(\text{IP}\right)}, \end{array}$$

where *N* is the total number of pixels for an image (explanations and real masks). IP is the set of pixels with the highest contribution values in a certain top percent [e.g., 1, 5, 10%, should be less than $\frac{Num\left(\text{RM}\right)}{N}$] in explanations. RM is the set of pixels labeled in corresponding real masks. *Num*(·) is the function to count the number of elements in a set. Higher PIP values indicate that more key pixels from the explanations are labeled in the real mask.

## 3. Materials and Results

### 3.1. Data and Three Tasks

Image classification is a common task in forestry. To verify this study, we designed three tasks: distinguishing between real leaves and simulated data (binary classification), identifying diseased leaves (binary classification), and classifying plant species (multiclass classification), based on the open-source *PlantVillage* dataset (Hughes and Salathé, 2015). *PlantVillage* dataset contains leaf images of multiple plant species and also has labels for each sample such as healthy and diseased. It has been used in many studies on plant disease identification (Mohanty et al., 2016; Geetharamani and Pandian, 2019; Abade et al., 2021).

#### 3.1.1. Distinguishing Between Real and Simulated Data

**Figure 4:**
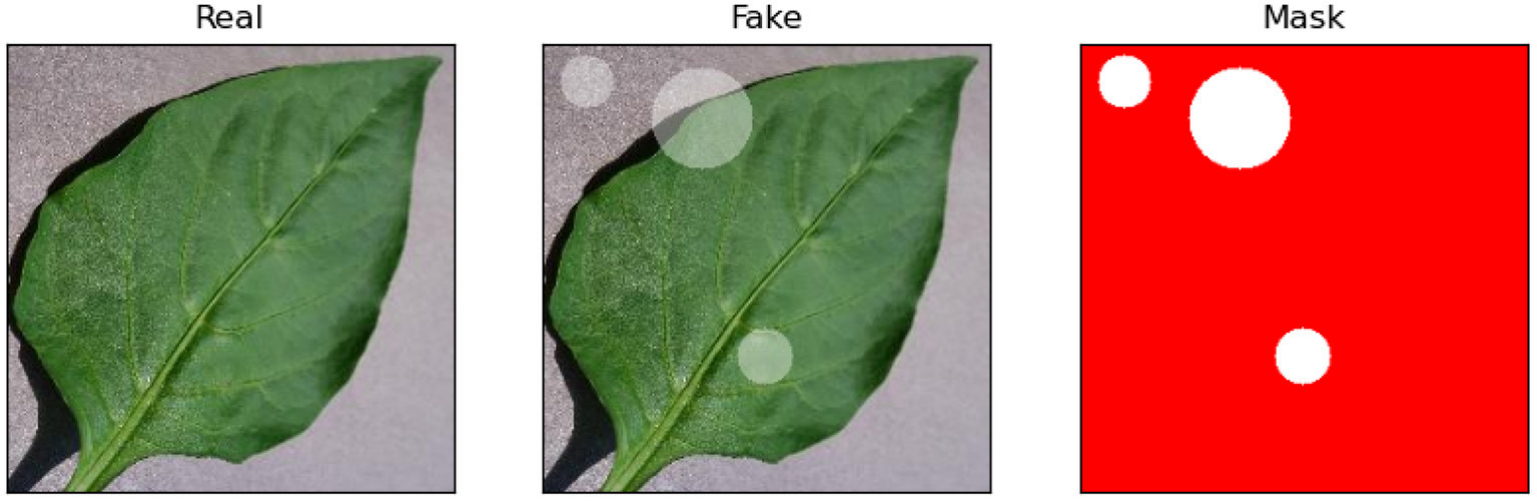
Simulated leaves generation. The labeled pixels in the real mask are red.

This study aims to highlight that the expertise can improve the training of deep learning models and make the explanations of models more similar to the predetermined annotation matrix (i.e., the real mask). But for a specific task, it is difficult to assess explanations fairly due to the human errors in generating the corresponding real mask of each input sample. Therefore, we simulated images with definite real masks. The specific way of simulated data generation is to select a few images of healthy pepper leaves and then randomly add some transparent circles (number, size, and location are random) into the leaf (as displayed in Figure 4). The real masks of generated images are the pixels outside the added circles. The purpose of adding transparent circles is to simulate the thinning of diseased leaves. The training objective of this experiment is to distinguish between real pepper leaves and fake leaves. To increase the difficulty of the task, added circles are allowed to be located in the background of simulated images.

The total number of samples (half real half fake) in this experiment is 2956. Train data, validation data, and test data contain 1773 (60%), 591 (20%), and 592 (20%) samples, respectively. The training process was implemented based on the PyTorch framework.^5^ The applied network is AlexNet (Krizhevsky et al., 2012), a well-known network in computer vision tasks. It is noted that we choose AlexNet as an example and do not consider other known networks. Since this research focuses more on model improvement based on expertise rather than absolute classification accuracy. Besides, for better comparability between the results, we used the same network structure in all three experiments, with the only alteration being the number of neurons (2 or 10) in the output layer. For the task of distinguishing between real and fake leaves, we trained two models with the same number of epochs (i.e., 90) and got two explanations for each input using the Grad-CAM method (Selvaraju et al., 2017). The RRR method (Ross et al., 2017) was only applied in the second training process, which means that the second model considers the specific expertise provided by the real masks. The weight λ of the RRR loss (Equation 5) was 1.5 in this experiment.

#### 3.1.2. Identifying Diseased Leaves

**Figure 5:**
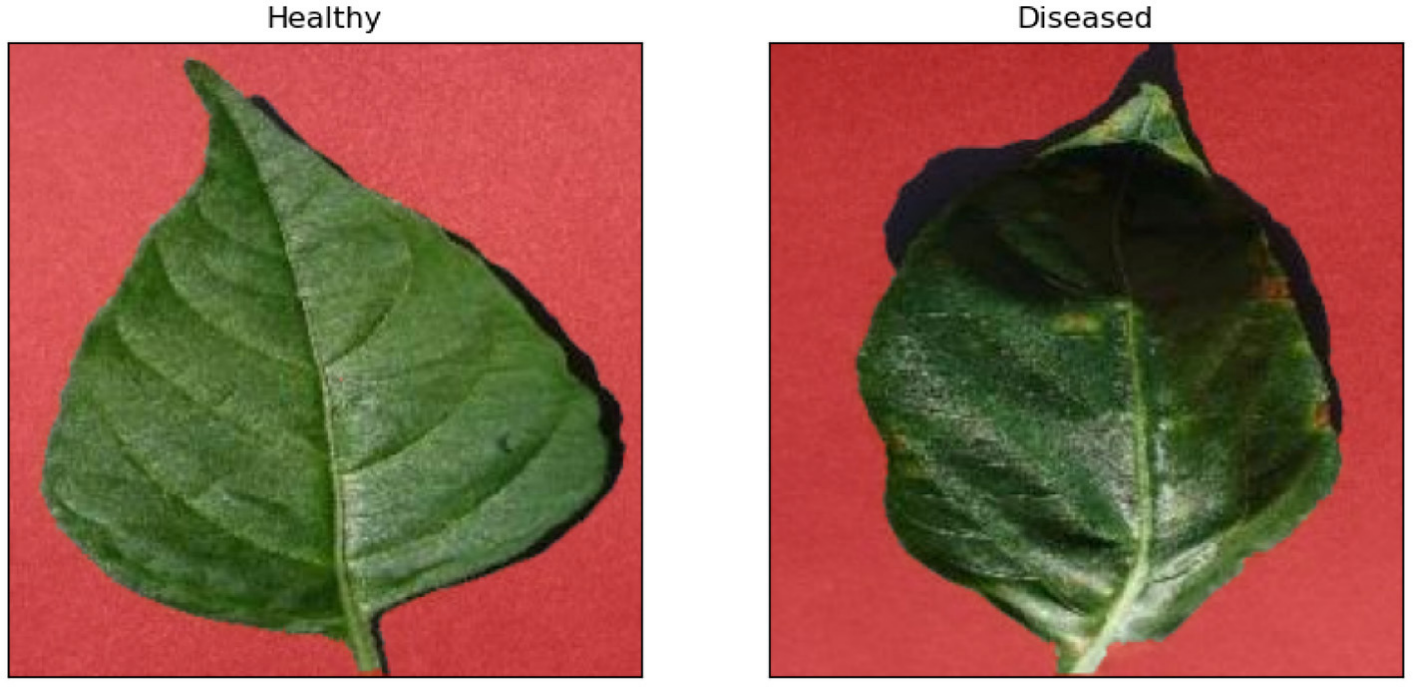
Examples of the real masks of the healthy leaf and the diseased leaf. The labeled pixels in masks are red.

Identifying diseased samples is a common task in forestry. This experiment aims to prove that the expertise and explanations can improve the deep learning models trained for the identification of diseased pepper leaves. The total number of image samples is 1994, including 997 images of healthy leaves and 997 images of diseased leaves. We divided the samples into three parts for the model training, which are the train data (1196 samples, 60%), validation data (399 samples, 20%), and test data (399 samples, 20%). The network structure for this experiment is the same as the first experiment (i.e., AlexNet). The difference between this experiment and the simulation experiment is difficult to mark the key pixels for the diseased leaf identification. However, for the RRR method, it is sufficient to label the assuredly useless pixels. Therefore, we separated the background pixels of each sample using the GrabCut algorithm (Rother et al., 2004) and labeled these pixels as the real masks. Figure 5 displays two such examples. We trained two deep learning models, similar to the previous simulation experiment. The number of epochs is 60 for both training processes and the expertise in annotation matrix form was only used for the second training. The weight λ of the RRR loss (Equation 5) was 2 in this experiment.

#### 3.1.3. Classifying Plant Species

**Figure 6:**
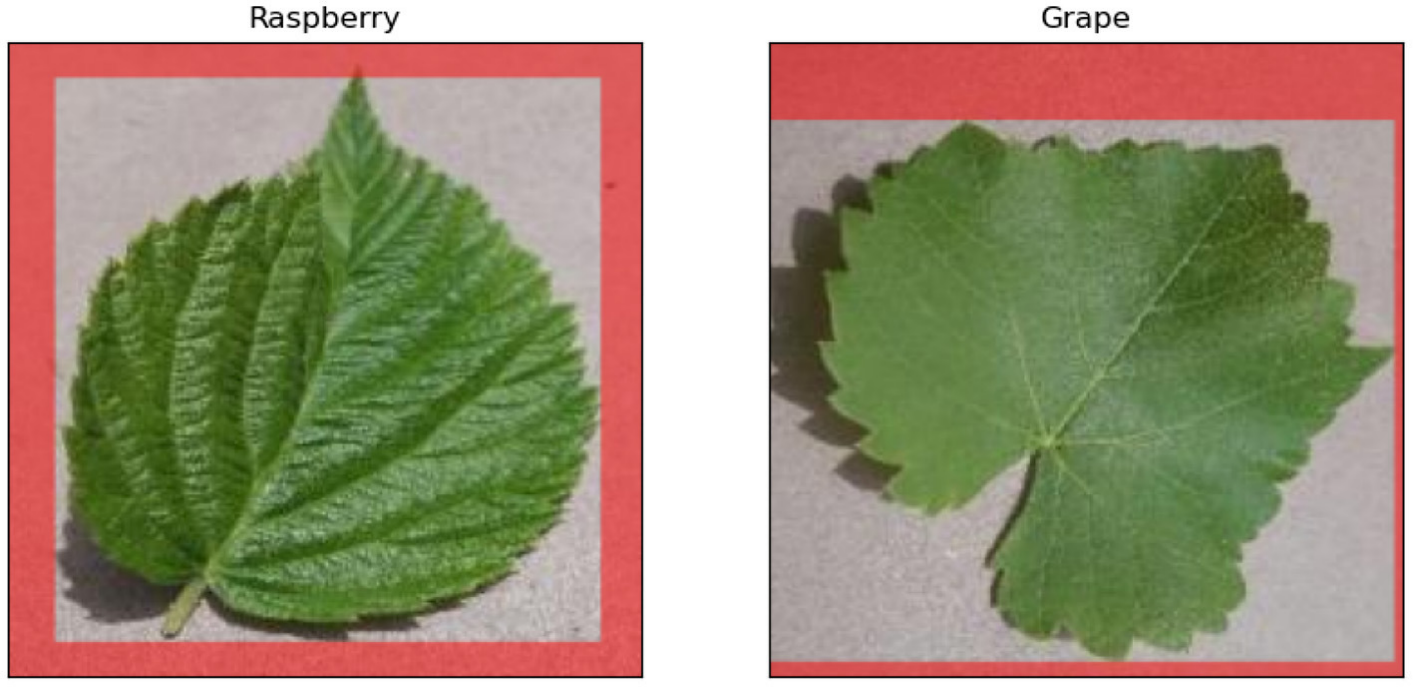
Examples of the real masks of two species' leaves. The labeled pixels in masks are red.

This experiment trains deep learning models to classify plant species, which is also a common task in forestry. We selected leaf images of 10 plant species, namely cherry, peach, potato, soybean, strawberry, raspberry, tomato, blueberry, apple, and grape. Compared with the previous two binary classification tasks, this multiclass classification is more complex. The total number of samples in this experiment is 1520, and each plant species has the same number of samples (i.e., 152). To train the model, we randomly divided the samples into three parts: train data (912 samples, 60%), validation data (304 samples, 20%), and test data (304 samples, 20%). The network structure is identical to those of the previous two experiments (i.e., AlexNet), except that the number of neurons in the output layer is 10. The leaf shape is an important feature for species classification, unlike in the task of diseased leaf identification. Therefore, we labeled the pixels outside the minimum bounding rectangles of leaves as the real masks in this experiment, which retains the information of the leaves shapes. Figure 6 illustrates two examples of such masks. Similar to the previous two experiments, we trained two deep learning models and applied the RRR method in the second training. The weight λ of the RRR loss (Equation 5) was 2 in this experiment. The number of epochs is 90 for both training processes.

### 3.2. Results

**Figure 7:**
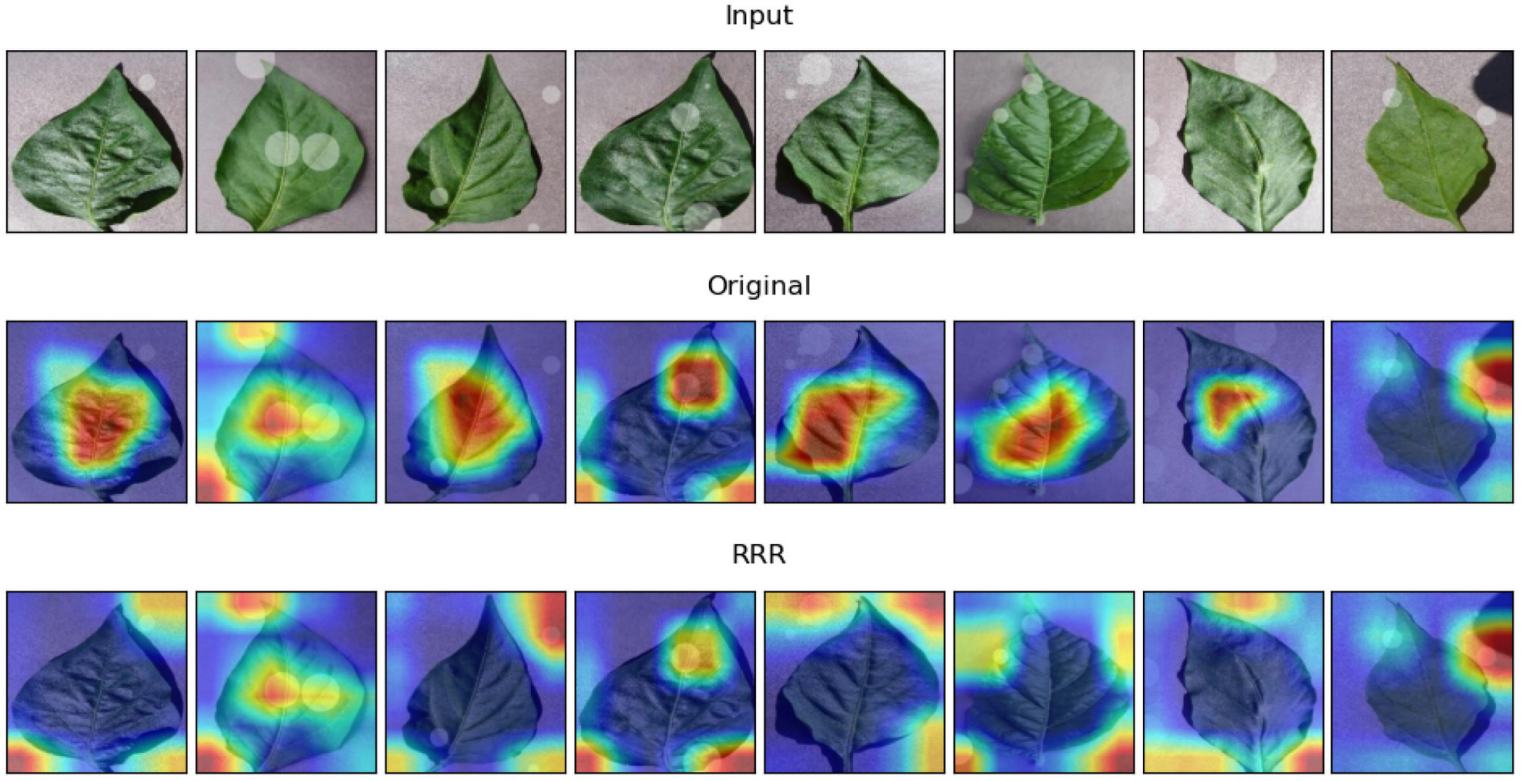
Examples of model explanations for the first task. The three rows display the input image, original explanation, and the explanation with the consideration of expertise. The pixels outside the added circles of each input are labeled as the real masks. A warmer color in the explanation indicates a higher contribution value, denoting a more important pixel for the classification task.

In terms of the first task, Figure 7 displays eight samples of input images and the two corresponding explanations. The quantitative results of the explanation assessment of the first task are given in Table 1. Figure 7 shows that many sample explanations are changed after incorporating expertise. The locations of pixels with higher contribution values typically shift from the center of leaves toward the added circles, which indicates that the model has gained more correct knowledge from the predetermined masks. The explanation assessment results in Table 1 show that all the indexes of new explanations (i.e., applying the RRR method) are better than those of the original explanations. Moreover, the classification accuracy has also increased 2.9% with the expertise, which is achieved while using the same train data, network structure, and training epochs. For the task of distinguishing between real leaves and simulated data, the results show that the consideration of expertise does indeed improve the deep learning model in terms of both accuracy and explanations.

**Table 1:** Accuracy and explanation assessment for the task of distinguishing between real and fake leaves.

**Models**	**Accuracy (%)**	**RMSE**	**CosineS**	**PIP**		
				**1**%	**5**%	**10**%
Original	81.6	0.583	0.440	60.3	59.6	58.2
RRR	**84.5**	**0.580**	**0.462**	**61.2**	**60.7**	**58.9**

*PIP is calculated based on three certain percents: 1, 5, and 10%*.

*The better results of every index are in bold*.

**Figure 8:**
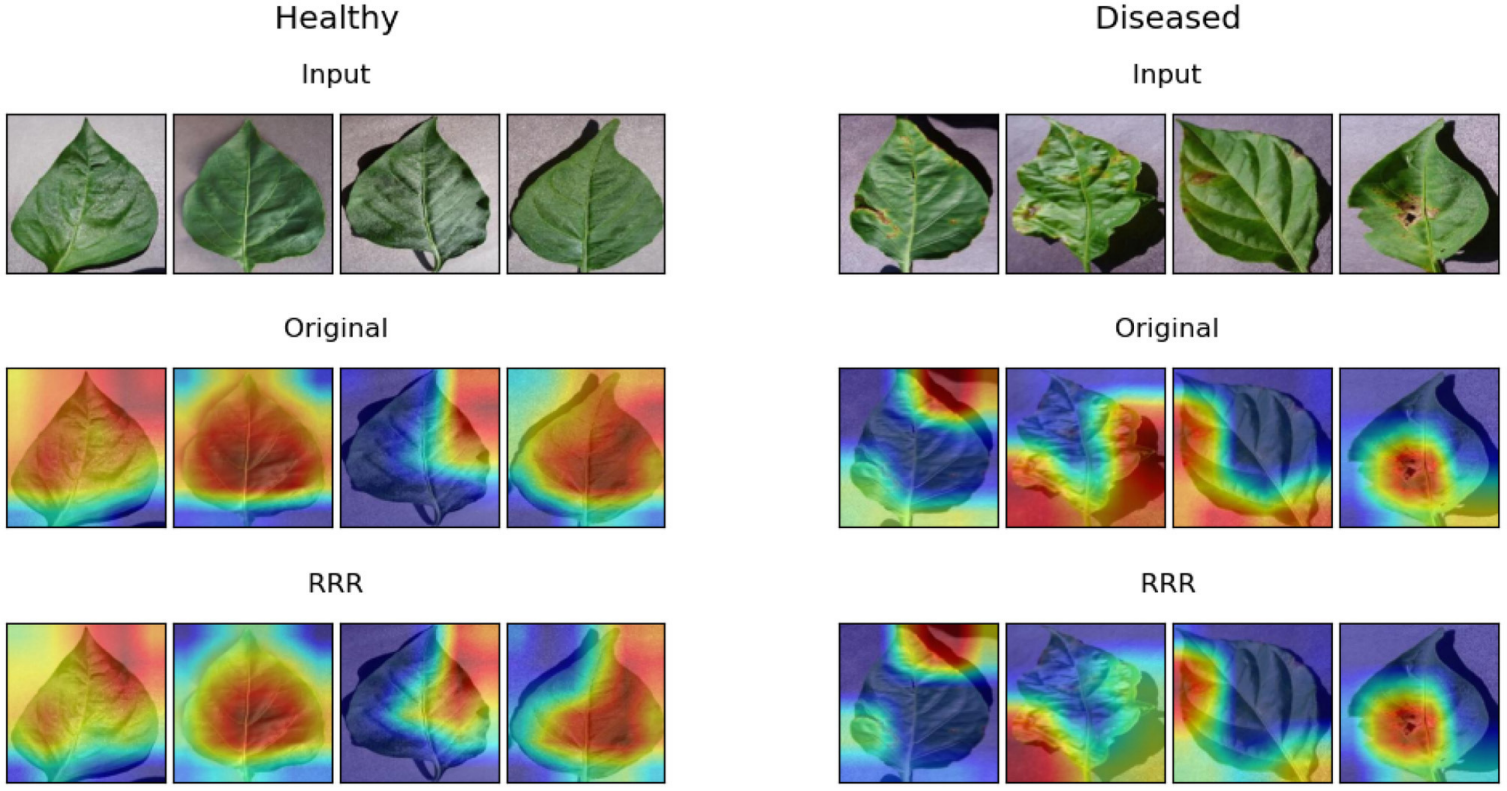
Examples of model explanations for the second task. The two columns reflect the explanations of healthy leaves and diseased leaves. For each column, the three rows display the input image, original explanation, and the explanation with the consideration of expertise. A warmer color in the explanation indicates a higher contribution value, denoting a more important pixel for the classification task.

In terms of the second task, Figure 8 displays the examples of eight samples' explanations (four healthy leaf samples and four diseased leaf samples) obtained by the Grad-CAM method. The explanations of the two trained models look similar, but it can be seen that with the utilization of the RRR method, the warm pixels appear less at the corners of the image in the corresponding explanations, especially for the second and third examples of diseased leaves. It proves that the second trained deep learning model has been driven to ignore corner background pixels according to the predetermined masks. Table 2 shows the results of model accuracy and explanation assessment for the task of identifying diseased leaves. The classification accuracy and all three explanation assessment indexes of the second trained model improve on the original ones. The slight improvement in classification accuracy (0.02%) maybe due to the already high original accuracy (>95%). It may also be caused by the simplicity of the real masks, i.e., labeling the useless background pixels, which leverages limited expertise. Nevertheless, the results of this experiment prove that it is possible to improve the deep learning models of identifying diseased leaves.

**Table 2:** Accuracy and explanation assessment for the task of identifying diseased pepper leaves.

**Models**	**Accuracy (%)**	**RMSE**	**CosineS**	**PIP**		
				**1**%	**5**%	**10**%
Original	95.5	0.533	0.710	61.4	62.2	63.8
RRR	**95.7**	**0.530**	**0.714**	**66.2**	**65.7**	**65.8**

*PIP is calculated based on three certain percents: 1, 5, and 10%*.

*The better results of every index are in bold*.

**Figure 9:**
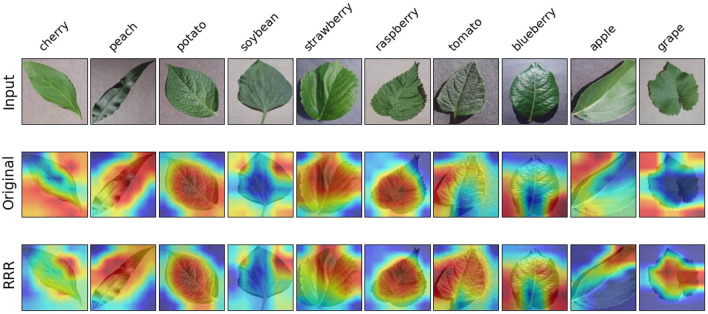
Examples of model explanations for the third task. The 10 columns reflect the explanations of 10 species' leaves. The three rows display the input image, original explanation, and the explanation with the consideration of expertise. A warmer color in the explanation indicates a higher contribution value, denoting a more important pixel for the classification task.

In terms of the last task, Figure 9 illustrates examples of model explanations for each of the 10 plant species. With the expertise in annotation matrix form, the trained model focuses more on the center pixels, pertaining to the leaf rather than the corners, as can be seen obviously in the apple and grape samples, which is analogous to the explanation improvement in the experiment of identifying diseased leaves. Additionally, the model with RRR utilization has an increased focus on the leaf edges (e.g., the cherry sample in Figure 9), which is consistent with common sense. Table 3 provides the results of model accuracy and explanation assessment for the task of classifying plant species. The second model surpasses the first model in both accuracy and explanation assessment indicators. The improvement in classification accuracy (4.6%) is the largest among all three experiments, despite labeling a relatively small number of useless pixels (as displayed in Figure 6) in the masks. The results of this experiment show that it is possible to improve the deep learning models for complex tasks.

**Table 3:** Accuracy and explanation assessment for the task of classifying plant species.

**Models**	**Accuracy (%)**	**RMSE**	**CosineS**	**PIP**		
				**1**%	**5**%	**10**%
Original	87.8	0.563	0.797	81.1	82.6	83.6
RRR	**92.4**	**0.550**	**0.810**	**86.1**	**87.3**	**87.3**

*PIP is calculated based on three certain percents: 1, 5, and 10%*.

*The better results of every index are in bold*.

The consideration of model explanations and corresponding expertise can improve deep learning models in forestry, as demonstrated by the three experiments. The degree of model improvement is directly related to the task difficulty and quality of the expertise.

## 4. Discussion

Deep learning models require mining task-related knowledge from the data. But for some practical applications, it is difficult to avoid outliers in the train data. The outliers will affect the model training because they contain the wrong information for the task. However, the new research framework proposed in this study can reduce such impact. Based on this framework, sample-based explanations can be obtained by using XAI methods. The corresponding explanations of outlier data may be different from other normal samples' explanations, which helps identify outliers and remove them from the train data. Moreover, as mentioned in Section 2.3, the applied FUL method RRR does not require labeling the annotation matrix of all samples. It means that the corresponding real masks of potential outlier data can be set as a zero matrix, which has no additional impact on model training.

The sampling variability could also affect the performance of deep learning models. To verify that the proposed framework is robust to the train data, we take the third task (i.e., classifying plant species) as an example and use the five-fold cross-validation method. The original data are divided into five equal parts. For each experiment, four of them form the train data, while the other one is used for testing. All the network parameters and experimental processes are the same as the ones in the above experiment (Section 3.1.3). Table 4 provides the results of model accuracy and explanation assessment (take RMSE as an example) in the five-fold cross-validation. The max and min values of accuracy and RMSE are close, which proves that the model performance is stable for different train data. Besides, the models using RRR surpass the original models in both classification accuracy (the average, max, and min values of classification accuracy) and explanation assessment (the average and max values of RMSE). The result verifies that this study is robust to sampling variability.

**Table 4:** Accuracy and explanation assessment (RMSE) results for the five-fold cross-validation.

**Models**	**Average_A (%)**	**Max_A (%)**	**Min_A (%)**	**Average_R**	**Max_R**	**Min_R**
Original	89.3	90.1	87.5	0.564	0.582	**0.550**
RRR	**90.2**	**92.4**	**89.1**	**0.561**	**0.571**	0.552

*Average_, Max_, and Min_ denote the average, max, and min values of the corresponding indicators (i.e., accuracy and RMSE) in five experiments*.

*The better results of every index are in bold*.

## 5. Conclusions

This research aims to improve deep learning models in forestry based on model explanations and corresponding expertise. Based on the review of relevant studies on deep learning applications in forestry, XAI methods, and FUL methods, we proposed a new research framework which includes consideration of explanations and expertise produces a reliable model in actual tasks. To prove our point, we designed and performed three experiments for various training tasks based on plant leaf data. The qualitative and quantitative comparison of accuracy and model explanations shows that the predetermined annotation matrices (i.e., expertise) can guide and improve deep learning models. For all three experiments, the classification accuracy is increased (up to 4.6% in a 10-class classification task) when considering expertise, and the improvement in model explanation is also reflected by three indexes of explanation assessment (i.e., RMSE, CosineS, and PIP). Besides, we also discussed the impact of outlier data and sampling variability on this study.

This research highlights the important role of model explanations and expertise for deep learning studies in forestry, especially with the growing impact of artificial intelligence and big data and the ever-increasing utilization of deep learning methods in this field. Furthermore, it serves as a reference for relevant studies. It should be mentioned that the masks we used were relatively simple, therefore we can expect the deep learning models to have an even greater improvement with higher quality expertise. Our experiments consisted entirely of image classification tasks in this study. The idea of using explanations and expertise to improve deep learning models can also be applied in other tasks such as time-series forecasting; all that is required is to utilize the available XAI and FUL methods, or design new ones. We intend to extend the application scenarios in the future.

## Data Availability Statement

The data and codes of this study are available in [github.com] with the link (https://github.com/adoosth/xaiforestry), further inquiries can be directed to the corresponding author.

## Author Contributions

XC leaded the conception and design of the study, found the opensource dataset, and wrote the first draft of the manuscript. JK contributed to the conception and particularly the data augmentation. XC, AD, and JK designed the experiments. AD processed the data and conducted experiments. All authors contributed to manuscript revision, read, and approved the submitted version.

## Funding

This research was funded by the FORESTCARE project under the Digital GreenTech (no. 02WDG014E), Federal Ministry of Education and Research (BMBF), Germany.

## Conflict of Interest

The authors declare that the research was conducted in the absence of any commercial or financial relationships that could be construed as a potential conflict of interest.

## Publisher's Note

All claims expressed in this article are solely those of the authors and do not necessarily represent those of their affiliated organizations, or those of the publisher, the editors and the reviewers. Any product that may be evaluated in this article, or claim that may be made by its manufacturer, is not guaranteed or endorsed by the publisher.

## Footnotes

^1^Sustainable Development Goals: https://sdgs.un.org/goals.

^2^Clever Hans: https://en.wikipedia.org/wiki/Clever_Hans.

^3^Root-mean-square error: https://en.wikipedia.org/wiki/Root-mean-square_deviation.

^4^Cosine similarity: https://en.wikipedia.org/wiki/Cosine_similarity.

^5^PyTorch: https://pytorch.org/.
